# Regulation of the 26S Proteasome: From Homeostasis to Stress and Disease

**DOI:** 10.3390/cells15141247

**Published:** 2026-07-10

**Authors:** Victoria Cohen-Kaplan, Aaron Ciechanover, Yelena Kravtsova-Ivantsiv

**Affiliations:** Rappaport-Technion Integrated Center (R-TICC) and the Rappaport Faculty of Medicine and Research Institute, Technion-Israel Institute of Technology, Haifa 3109601, Israel; yelenaiv@technion.ac.il

**Keywords:** 26S proteasome, cellular stress, ubiquitin, nucleus, cytosol, liquid–liquid phase separation (LLPS), biomolecular condensates

## Abstract

**Highlights:**

**What are the main insights?**

- The 26S proteasome is a dynamically regulated proteolytic complex, the activity of which is controlled by subunit and subcomplex composition, post-translational modifications, and spatial organization.
- Emerging regulatory mechanisms have reshaped the conventional view of the proteasome as a passive degradation machinery, revealing its role as a dynamically regulated determinant of cellular proteostasis.

**What are the implications of the main findings?**

- Proteasome remodeling enables cellular adaptation to metabolic and stress conditions, whereas disruption of these regulatory mechanisms contributes to disease pathogenesis.
- Understanding the mechanisms governing proteasome regulation provides new insights into the maintenance of proteostasis and identifies potential therapeutic opportunities for proteostasis-related diseases.

**Abstract:**

The ubiquitin–proteasome system (UPS) has traditionally been described as a tightly regulated degradative network driven mainly by the specificity of its ubiquitin-conjugating enzymatic components. The 26S proteasome is the catalytic arm of the system that acts downstream to the conjugation machinery. For a long time, it has been considered to be a constitutive multi-subunit proteolytic complex that recognizes in a non-discriminatory manner ubiquitin-marked target substrates with less than a handful of exceptions. However, emerging evidence reveals that the 26S proteasome function is also dynamically regulated by multiple factors, such as subunit composition and synthesis, post-translational modifications, and spatial localization, all of which are tightly regulated by the metabolic and stress states of the cell. Importantly, dysregulation of these newly emerging regulatory mechanisms has pathogenic sequelae. These mechanisms fine-tune proteasome activity and expand its role as an active regulator of protein homeostasis rather than being a passive degradation machinery. Given the rapid expansion of these findings and their impact on our understanding of proteasome biology, an integrated overview of these regulatory mechanisms is timely.

## 1. Introduction

Cellular homeostasis depends in part on maintaining a delicate equilibrium between the synthesis and selective degradation of proteins. This highly energy-demanding balance is of critical importance, as its dysregulation can lead to a broad spectrum of diseases [1]. Protein degradation serves several key functions: (i) maintaining protein quality control (PQC) by removing misfolded or damaged proteins that are toxic for cellular homeostasis [2]; (ii) eliminating regulatory proteins, such as transcription factors and cell cycle inhibitors, whose activity must be restricted to defined time windows [3,4]; (iii) converting inactive newly synthesized proteins into their active counterparts via their partial degradation or cleavage [5]; (iv) generating peptides for innate and adaptive immune response, as well as for neuronal signaling [6]; and (v) enabling rapid cellular adaptation under stress conditions [7,8]. The vast majority of these processes are carried out by the UPS.

Ubiquitination is a three-step enzymatic process. First, a ubiquitin-activating enzyme (E1) forms an energy-dependent thioester between a cysteine residue in its active site and the C-terminal glycine of ubiquitin (Ub). The activated Ub is then transferred to an Ub-conjugating enzyme (E2). From E2, Ub can be transferred directly to the substrate that is bound specifically to an Ub ligase–Ring finger E3s. Alternatively, it can be transferred to the substrate via a thioester intermediate on the ligase–HECT domain E3s. The C-terminus of the first Ub moiety is attached covalently to an ε-NH_2_ group of an internal Lysine (Lys, K) residue in the target protein. The second and following Ub moieties can be attached to one of seven Lys residues in the previously conjugated moiety to generate an Ub chain. Lys48-linked polyUb chains serve as the most common signal for targeting the protein for degradation by the 26S proteasome—the core proteolytic component of the system [9]. Recent studies, however, reveal a much broader diversity of Ub signals: the proteasome can recognize chains with alternative internal linkages, linear (head-to-tail rather than branched) or mixed Ub/Ub-like conjugates, and even substrates modified by mono- or multi-monoubiquitinations [10,11]. Mammalian cells possess mostly a single E1 enzyme, a few dozens of E2s [12], and hundreds of E3 ligases [13] that operate together in a hierarchical cascade to modify simultaneously numerous substrates.

In addition to the proteasome [14], a second major degradation pathway in eukaryotic cells is the autophagy–lysosome pathway (ALP), which eliminates—mostly in a Ub-dependent manner—large protein aggregates and dysfunctional organelles, including proteasomes, under various stress conditions [15].

While the original thought was that the proteasome is a protease that recognizes specifically ubiquitinated targets but has no additional layers of regulation, this picture has changed dramatically in recent years. It has been shown that different modes of post-translational modifications affect different proteasomal subunits, which, along with changes in subunit composition and even subcomplexes, and more recently in its subcellular compartmentalization, regulate its activity. Importantly, these regulatory mechanisms are not static but are continuously remodeled in response to physiological cues and are frequently disrupted in disease. Consequently, proteasome dysregulation emerges as a central determinant of proteostasis failure across a variety of pathological conditions.

**Figure 1:**
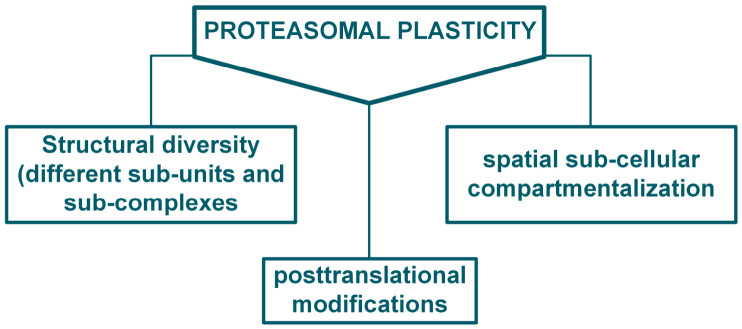
Schematic diagram illustrating the regulation of proteasomal function through different mechanisms.

Disease states provide a functional context in which the complexity of proteasome regulation becomes particularly evident, revealing how disruptions in proteasome dynamics contribute to the loss of proteostasis and disease pathogenesis. The main aim of this review is to summarize recent advances in our understanding of how structural, post-translational, and spatial regulation govern proteasome function in health, stress adaptation, and disease (Figure 1).

## 2. Structural Diversity and Post-Translational Modifications Regulating Proteasomal Function

**Figure 2:**
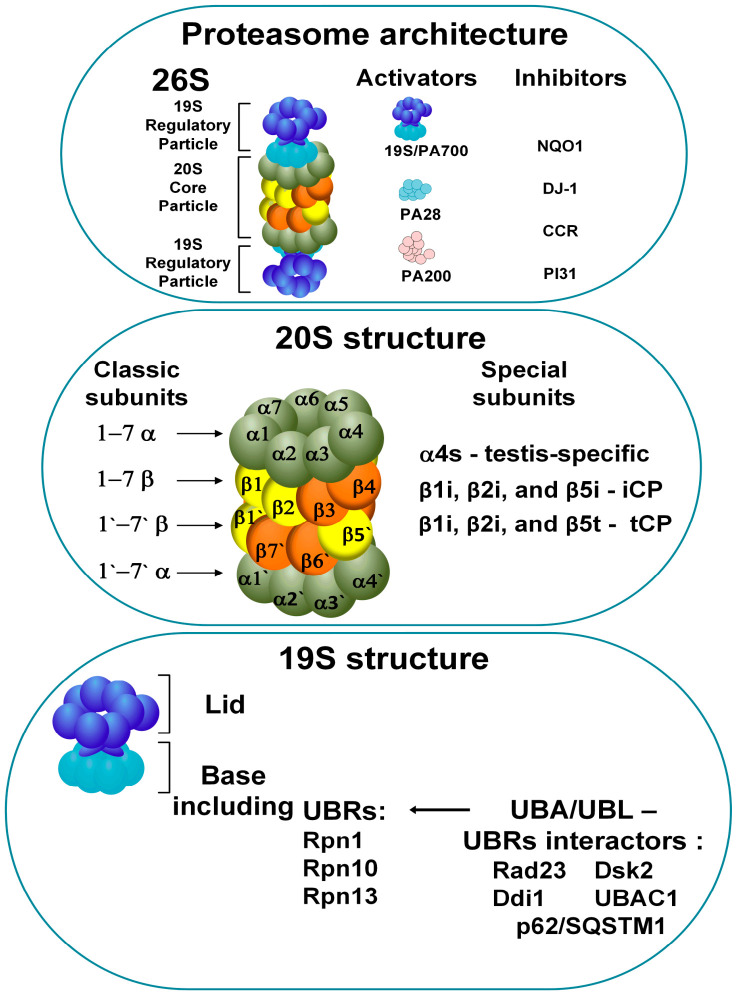
Schematic representation of proteasomal architecture. Proteasomes consist of a 20S core particle (CP) that catalyzes proteolysis and can be capped on one or both ends by regulatory particles (activators), including 19S (PA700), PA28, or PA200. Proteasomal activity can be modulated by factors such as NQO1, DJ-1, CCR, and PI31. The canonical 20S CP consists of two inner β-rings flanked by two outer α-rings. Alternative forms of the 20S proteasome have been described, including the testes-specific proteasome, immunoproteasome (iCP), and thymoproteasome (tCP), as indicated. The 19S regulatory particle consists of a “lid” and a “base” subcomplexes. Several base subunits, including Rpn1, Rpn10, and Rpn13, function as intrinsic Ub-binding receptors (UBRs). In addition, UBA/UBL domain-containing proteins act as extrinsic ubiquitin-binding receptors that recruit ubiquitinated substrates to the proteasome.

Proteasome regulation is underpinned by its structural plasticity and compositional diversity (Figure 1 and Figure 2), which enable the formation of functionally distinct assemblies adapted to different cellular demands. Post-translational modifications further refine proteasome activity by modulating its stability, assembly, and catalytic function. Together, these mechanisms define the core regulatory principles underlying proteasome adaptability under physiological conditions.

### 2.1. Dynamic Assembly and Composition of Proteasome Complexes

The proteasome holoenzyme, one of the most structurally and functionally complex multi-subunit enzymes in eukaryotic cells, was initially discovered in the early 1980s [16,17,18,19]. Fulfilling the final step in the cascade of reactions of the UPS, it possesses a dynamic structure (Figure 2) that provides the very last layer of recognition of substrates destined for degradation. The main core particle (CP) responsible for catalyzing the proteolytic reactions is the 20S hollow barrel, which exhibits trypsin-, chemotrypsin-, and caspase-like catalytic activities. This complex consists of an inner double-heptamer β-ring (β1–β7x2 subunits) flanked by two outer heptamer α-rings (α1–α7x2 subunits). In the active state, the 20S catalytic complex is capped by one or two regulatory particles (RPs) that act as a “lock pick” which lifts—in an energy-dependent manner—the interlocking tails of the α subunits that hinder entry into the catalytic β-ring chamber [20].

Several alternative structures of the CP have been characterized. For instance, one form contains the testis-specific α4s subunit, which replaces the standard α4 subunit in spermatocytes [21,22], while another features the substitution of α3 with α4, resulting in a constitutively open β-ring [23]. Although the latter type of proteasome highlights structural plasticity, its existence in nature and mostly its function remain controversial. CPs with alternative β subunits, the immunoproteasome (iCP) and the thymoproteasome (tCP), play important roles in adaptive immunity. In iCP, the catalytically active subunits β1, β2, and β5 are substituted by β1i, β2i, and β5i, yielding peptides that are better presented on the Major Histocompatibility type I (MHC I) Complex [24,25]. tCP proteasome, in turn, harbors β1i, β2i, and β5t subunits with reduced chymotryptic activity, producing peptides depleted of hydrophobic C-terminal residues. tCP is found in cortical thymic epithelial cells and plays a role in immune self-discrimination [26,27]. These variations in the CP enable fine-tuning of proteasomal catalytic activity to meet specific tissue requirements.

Various exchangeable RPs with distinct functional properties have been identified in eukaryotic cells. The most studied and frequently occurring RP is the 19S (also known as PA700), which, when bound to the 20S core, forms the “canonical” 26S proteasome. The 19S RP consists of “base” and “lid” subcomplexes, each composed of multiple subunits [28]. The “lid” mediates recognition of Ub chains and exhibits deubiquitinating activity, while the “base” possesses ATPase activity and is required for binding, unfolding, and translocation of substrates. The recruitment of ubiquitinated substrates to the proteasome is mediated by direct binding to the intrinsic, “built-in” UBRs Rpn1, Rpn10, and Rpn13 [29,30]. Substrate delivery can also occur through an additional layer of regulation mediated by shuttle proteins, such as the ubiquitin-associated (UBA) and ubiquitin-like (UBL) domain-containing proteins Rad23, Ddi1, Dsk2, UBAC1, and p62/SQSTM1, which bind to UBRs [30,31,32,33,34]. Crystal structures of Rad23-Rpn1 and PLIC2-Rpn13 complexes evidently show that each shuttle protein possesses a distinct UBL domain structure, providing unique binding preferences for specific intrinsic UBRs [35]. In another study, competition between Rpn1 and Rpn10 for Dsk2 binding was demonstrated, highlighting some redundancy among UBRs in the proteasome [36].

Proteasome assembly and maturation are coordinated by dedicated chaperones, including UMP1/POMP, which are essential for 20S core particle biogenesis and ensure proper maturation of functional proteasomes by preventing the accumulation of incomplete or inactive assembly intermediates [37].

The 26S proteasome ensures the turnover of key factors in cellular regulation in an ATP- and Ub-dependent manner, thereby controlling a broad range of dynamic cellular processes such as cell cycle, signal transduction, apoptosis, and stress responses. Recent structural and proteomic studies have demonstrated the plasticity of the 19S RP, which varies in a conformation- and compartment-specific manner to meet dynamic cellular requirements. For example, high-resolution cryo-electron microscopy analyses have revealed that the redox-active cofactor TXNL1 interacts with the 19S RP dependent on the proteasome’s conformation and activity state. When the proteasome is inactive, TXNL1 binds with low affinity to several sites of Rpn2, Rpn10, and Rpn11 subunits. In contrast, during active substrate degradation, TXNL1 covers the catalytic groove of the Rpn11 deubiquitinase (DUB) and coordinates the active-site Zn^2+^, thereby modulating its activity [38]. Rpn11, along with the transiently associated DUBs–Ub-specific peptidase 14 (USP14) and Ub carboxyl-terminal hydrolase isozyme L5 (UCH-L5), represents the main deubiquitinating activity of the proteasome [39,40,41]. However, a study employing in situ cross-linking mass spectrometry revealed the transient association of an additional DUB enzyme, Ub-specific peptidase 15 (USP15) [42]. Moreover, the same group demonstrated that the translation initiation factor EIF3M binds to the 19S RP, replacing the canonical Rpn9 subunit and giving rise to a hybrid form of the proteasome [42]. In recent years, the application of new techniques has revealed transient and possibly auxiliary binding partners, supporting the view that the 19S RP facilitates both permanent and dynamic protein interactions to fine-tune proteasomal function.

Other RPs that function independently of ATP and Ub include PA28 (11S), which facilitates the degradation of peptides, as well as oxidized, damaged, or unfolded proteins [43,44], and PA200, which mediates the degradation of acetylated histones in the nucleus [21,45]. Recently, it was shown that proteasome composition and cleavage activity are remodeled upon bacterial infection, particularly through recruitment of the PSME3 (PA28g) RP, which enhances tryptic-like peptide cleavage. This process generates cationic peptides that directly disrupt bacterial membranes, providing a cell-autonomous innate immunity defense, independently of the classical antigen presentation route [46].

In addition to activators, inhibitors of the 20S proteasome have also been identified. For example, NAD(P)H dehydrogenase [quinone] 1 (NQO1) interacts with the tumor suppressors p53 and p73, as well as with the 20S proteasome, protecting tumor suppressors from degradation [47]. The level of NQO1 is tightly regulated by the concentration of flavin adenine dinucleotide (FAD), indicating that proteolysis by the proteasome is linked to the metabolic state of the cell [48]. DJ-1 is another inhibitor of the 20S proteasome that rescues α-synuclein and p53 from degradation and regulates NQO1 levels under oxidative stress [49]. A bioinformatic screen based on the known proteasome regulators revealed a novel family of 20S inhibitors, termed catalytic core regulators (CCR). These small 20-30 kDa proteins bind exclusively to 20S and not the 26S, inhibiting the degradation of proteins both in vitro and in cells, preferentially under oxidative stress [50]. The proteasome inhibitor subunit 1 (PI31) represents an interesting regulator of 20S proteasome activity. Cryo-electron microscopy (cryo-EM) studies have shown that the PI31 protein interacts directly with the catalytic domain of the 20S via two copies of its disordered C-terminus, adopting a conformation that makes it resistant to degradation and blocks substrate proteolysis [51]. An interesting case of proteasome-dependent, however Ub-independent degradation is that of ornithine decarboxylase (ODC) [52,53] that requires for its degradation the “chaperoning” protein antizyme. Antizyme-1 associates with the ODC monomer, leading to exposure of the enzyme’s C-terminal domain for recognition by the 26S proteasome [54].

### 2.2. Post-Translational Modulation of Proteasome Function

Regulation of the proteasome life cycle, including assembly and autophagic degradation, as well as its localization and activity, strongly depends on a variety of post-translational modifications [55]. These include ubiquitination, SUMOylation, phosphorylation, oxidation, and proteolytic truncation, as well as N-terminal modifications such as acetylation, myristoylation, and methylation, among others [56,57].

Here, we address the regulation of distinct proteasome-associated events modulated by ubiquitination. For instance, specific ubiquitination of Rpt5 by the Ub ligase Not4 monitors chaperons’ activity, acting as a checkpoint during the formation of the ATPase Rpt ring. This prevents the assembly of incomplete or faulty complexes [58]. Under amino acid starvation, proteasomes undergo selective degradation (proteaphagy) via autophagy. This process is triggered by increased ubiquitination of Rpn2, Rpn10, and Rpn13, promoting recognition by autophagosomes. Conversely, the Rpn5, Rpn6, and α7 subunits display diminished ubiquitination. An interesting example involves the Rpn1 subunit, which, following starvation, undergoes ubiquitination on certain Lys residue(s) and reduction of ubiquitination on other(s), which probably serves to fine-tune the downstream effect of the modification [59].

Interestingly, proteasome activity is also regulated by ubiquitination of its subunits, which prevents their incorporation into the complex or their assembly with shuttle proteins. For example, Rpn10 was found to be ubiquitinated in its non-proteasomal, free state [60], markedly reducing its interaction with the shuttle proteins Dsk2 and Rad23 [61,62]. At the same time, the association of Dsk2 with the proteasome increases, indicating a mechanism that switches the proteasome between two states: high Rpn10/low Dsk2 and vice versa [61].

Monoubiquitination of several Lys residues in Rpn10 by the Rsp5/Ubp2 complex in yeast [63], as well as modification of its VWA domain in mammalian cells [64], together with ubiquitination of Rpn13, Rpt5, and Uch37, collectively impair substrate recognition, their deubiquitination, and subsequently their proteasomal degradation [63,65,66].

Modification by Ub moieties has been observed not only in subunits of the 19S RP but also in those of the 20S CP. In prostate cancer cell lines, ubiquitination of the α2 subunit of the 20S CP was reported to recruit δ-aminolevulinic acid dehydratase (ALAD) in place of the 19S RP, thereby inhibiting the chymotrypsin-like activity of the proteasome [67].

Altogether, these findings highlight ubiquitination as a versatile regulatory mechanism that, together with other modifications of proteasomal subunits, fine-tunes proteasome assembly, activity, and turnover, ensuring precise control of proteolysis under various cellular conditions.

## 3. Spatial Regulation of Proteasome Function: Localization, Translocation, and Compartmentalization

Proteasome activity is also governed by its spatial organization within the cell. Dynamic redistribution between nuclear and cytosolic compartments is driven by nutrient and stress signaling, generating functionally specialized proteasome pools. This spatial regulation is further integrated with biomolecular condensates and proteaphagy, linking localization to adaptive control of protein homeostasis.

### 3.1. Nuclear Import and Enrichment Mechanisms

In eukaryotic cells under nutrient-rich conditions, proteasomes are predominantly enriched in the nucleus, reflecting the vital role of regulated proteolysis in nuclear processes, including turnover of transcriptional regulators, cell-cycle factors, and chromatin-associated proteins. Possibly, the nucleus may also serve as a proteasome storage site [68,69,70].

This nuclear localization is an active, highly regulated process rather than a consequence of passive diffusion. Proteasome can enter the nucleus in multiple assembly states, including free subunits, assembly intermediates, and fully assembled 20S and 26S complexes. Proteasome nuclear import occurs through the nuclear pore complex (NPC) via importin/karyopherin pathways and associated adaptors. In budding yeast, the 19S RP base subcomplex contains basic nuclear localization signal (NLS)-like regions within the scaffolding subunit Rpn2, which engages the classical importin-α/β pathway (Kap60/Kap95) to facilitate nuclear import. Disruption of these signals in Rpn2 reduces nuclear localization of proteasomal base subcomplexes and increases their cytosolic accumulation [69,71]. Impaired nuclear assembly of the mature proteasome and defects in proteasome-mediated proteolysis support a model in which nuclear proteasomes are assembled from imported precursors. In addition to base-mediated import, alternative pathways facilitate nuclear entry of other proteasome species in yeast. The proteasome-associated factor Blm10 binds 20S core particles and promotes their nuclear import through interactions with phenylalanine-glycine (FG)-rich nucleoporins in a Ran-dependent manner. For fully assembled holoenzymes, adaptor proteins such as Sts1/Cut8 further contribute to nuclear translocation [72,73].

In mammalian cells, analogous mechanisms operate with distinct regulatory factors. AKIRIN2 functions as a key scaffold that binds mature proteasomes and recruits multiple importins to facilitate efficient nuclear import of the proteasome. Loss of AKIRIN2 disrupts nuclear proteasome localization and leads to accumulation of nuclear substrates (such as c-Myc), highlighting its essential role in nuclear proteostasis [74].

Additional regulators, such as PA200 and PA28γ, further modulate nuclear proteasome activity by stabilizing specific proteasome complexes and targeting them to defined subnuclear compartments [75,76].

Once inside the nucleus, proteasomes associate with chromatin and other subnuclear domains, positioning them to efficiently degrade substrates involved in transcriptional regulation, DNA repair, cell-cycle progression, and protein quality control [77,78,79].

### 3.2. Functional Specialization of Distinct Proteasome Pools

Proteasome compartmentalization underlies distinct functional specializations within the cell. Nuclear proteasomes primarily mediate the degradation of short-lived regulatory proteins that control transcription, cell-cycle progression, and DNA repair, including cyclins and transcription factors such as p53 and c-Myc [80]. The rapid turnover of these substrates enables tight temporal regulation of key nuclear processes, including checkpoint activation and transcriptional programs. In addition, nuclear proteasomes are spatially organized through associations with chromatin and subnuclear structures, such as promyelocytic leukemia (PML) bodies, nuclear speckles, and DNA damage foci, thereby facilitating localized substrate recognition and degradation at sites of active transcription and genome maintenance [81,82,83].

In contrast, cytosolic proteasomes predominantly degrade misfolded, damaged, or aggregation-prone proteins generated by translational errors, oxidative stress, or environmental challenges [84]. A specialized subset associates with the endoplasmic reticulum (ER) to support ER-associated degradation (ERAD), in which retro-translocated, polyubiquitinated lumenal proteins are processed to maintain proteostasis and prevent ER stress [85].

An interesting case is that of p53. While the tumor suppressor is active in the nucleus, it is probably degraded in the cytosol, as, in the nucleus, it is stabilized by Ub-specific peptidase 7 (USP7), its deubiquitinating enzyme [86,87].

The efficiency and specificity of proteolysis in these compartments are shaped by differences in substrate availability, local biochemical environments, and regulatory factors. Cytosolic proteasomes typically encounter an increased load of misfolded proteins under stress conditions, whereas nuclear proteasomes are more directly integrated into regulatory networks that govern gene expression and cell-cycle control.

### 3.3. Nuclear Export and Nutrient-Dependent Stress-Mediated Translocation

Proteasome localization is dynamically regulated through continuous nucleo-cytoplasmic trafficking that adapts to cellular conditions. In well-fed cells, the proteasome predominantly accumulates in the nucleus through a steady-state balance between nuclear import and export that is operational even under basal metabolic conditions. Pharmacological studies demonstrate the active nature of this process: inhibition of CRM1/exportin-1-dependent export by leptomycin B causes nuclear retention of the proteasome, whereas inhibition of importin α/β-mediated import by ivermectin shifts the proteasome to the cytosol [70]. These observations indicate that proteasome distribution is continuously adjusted in response to pathophysiological demands.

Cellular stress disrupts this equilibrium and promotes proteasome redistribution. It has been shown that in yeast, glucose/nitrogen starvation triggers a protective remodeling of the proteasome system: proteasomes exit the nucleus, dissociate into the 20S core and 19S RPs, and are sequestered into membrane-less cytoplasmic Proteasome Storage Granules (PSGs) [88,89,90,91,92]. Upon glucose re-feeding, PSGs rapidly dissolve, and proteasomes return to the nucleus, enabling swift recovery of proteolytic capacity [89,92,93].

Amino acid starvation in mammalian cells induces rapid CRM1/exportin-1-dependent nuclear export of the proteasome. This response is thought to support adaptation to nutrient stress by moving proteolytic activity to the cytosol, where degradation of intracellular proteins can supply recycled amino acids required for survival [70]. However, the exact molecular mechanisms coordinating basal and stress-induced proteasome trafficking have remained elusive.

**Figure 3:**
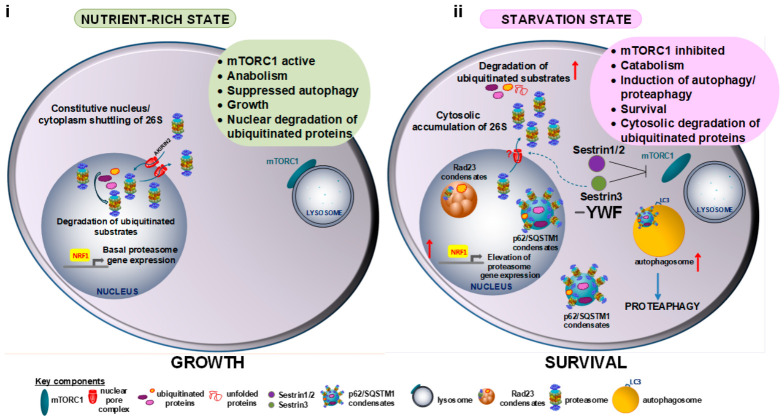
Integrated model of proteasome dynamics in nutrient-rich and stress conditions. The scheme summarizes how proteasome organization, localization, and function are remodeled in response to nutrient status and proteotoxic stress. (**i**) in nutrient-rich cells, the 26S proteasome is predominantly nuclear, supported by an active import pathway mediated by AKIRIN2 and “canonical” mTORC1 signaling. The 20S core particle associates with the 19S regulatory particle to mediate ATP- and ubiquitin-dependent protein degradation, while alternative activators such as PA28 and PA200 provide specialized functions. (**ii**) under amino acid starvation, mTORC1 activity is suppressed, including through aromatic amino acid sensing by Sestrin3, leading to proteasome redistribution from the nucleus to the cytosol. In the cytosol, proteasomes support increased protein turnover and amino acid recycling. Starvation and osmotic stress also promote the formation of p62/SQSTM1- and RAD23B-containing condensates (respectively) that concentrate ubiquitinated substrates and proteasomes, enhancing local proteolysis. Prolonged stress can trigger proteaphagy, in which proteasomes are selectively delivered to autophagosomes and lysosomes for degradation. Finally, NRF1-dependent transcription restores proteasome abundance after proteotoxic stress or starvation, thereby re-establishing proteostasis.

mTORC1 serves as a central regulator of this adaptive program by coupling nutrient availability to cellular metabolism. Under nutrient-rich conditions, mTORC1 is active at the lysosomal surface, where it promotes anabolic processes such as protein synthesis and suppresses catabolic pathways. Under these conditions, the proteasome is localized mostly to the nucleus. Amino acid deprivation inhibits mTORC1 signaling through Sestrin proteins and GATOR complexes, leading to dissociation of mTORC1 from lysosomal membranes and loss of downstream kinase activity. Consequently, protein translation is rapidly attenuated, metabolic programs are remodeled, and catabolic pathways, including autophagy and proteaphagy, are activated (Figure 3i,ii) [59,94,95,96,97]. Under these conditions, the large nuclear pool of the proteasome translocates to the cytosol.

A key advance has been the identification of amino acid-specific regulation of mTORC1 signaling. While leucine (Leu, L) and arginine (Arg, R) have long been known to activate mTORC1 through dedicated sensors, including the Leu sensors Sestrin1/2 and the Arg sensors CASTOR1 and SLC38A9 [98,99,100], recent studies reveal that aromatic amino acids—tyrosine (Tyr, Y), tryptophan (Trp, W), and phenylalanine (Phe, F)—uniquely modulate mTORC1 activity through Sestrin3 (Figure 3i,ii). thereby linking nutrient availability to proteasome dynamics [70]. Under YWF-limiting conditions, Sestrin3 reinforces inhibition of mTORC1 signaling through its interaction with the GATOR2 complex. In this context, deprivation of YWF induces proteasome nuclear export—a process initiated within a couple of hours and peaking approximately 4–6 hours after nutrient withdrawal. This redistribution enhances cytosolic proteolytic capacity, supporting adaptive remodeling of proteostasis during nutrient stress. The expanded cytosolic proteasome pool promotes degradation of non-essential cytosolic proteins, thereby generating recycled amino acids required to maintain cellular homeostasis and supporting cell survival [70].

Importantly, this process is reversible and reflects the dynamic nature of proteasome trafficking. Upon restoration of nutrient availability, mTORC1 signaling is reactivated, nuclear import pathways resume, and proteasomes gradually return to their predominantly nuclear localization [70]. This bidirectional trafficking highlights the plasticity of the proteasome system and its capacity to rapidly adapt to changing metabolic conditions.

**Figure 4:**
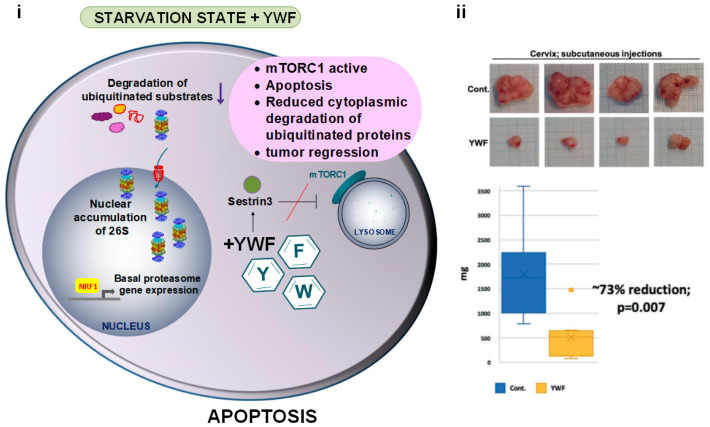
Proteasome dynamics under starvation conditions following addition of the three aromatic amin acids, Tyr, Trp, and Phe (YWF). (**i**) Under amino acid starvation, the suppression of mTORC1 is reversed by supplementation of the YWF, the triad of aromatic amino acids. YWF acts via Sestrin3, maintaining mTORC1 in an active state and preventing the proteasome from concentrating in the cytoplasm. As a result, instead of initiating a stress response, cells initiate the apoptotic program. (**ii**) HeLa cells were inoculated into mice to establish xenograft tumors. YWF was administered via subcutaneous injection directly into the tumor site. At the experimental endpoint, tumors were excised, weighed, and photographed on graph paper for scale. Tumor weights at the time of sacrifice are presented. Reprinted from Livneh et al., Cell Death and Differentiation (2024), with CC BY 4.0 license (https://creativecommons.org/licenses/by/4.0/).

Notably, it has been demonstrated that the addition of the YWF triad disrupts the inhibitory interaction between Sestrin3 and GATOR2, thereby enabling the reactivation of mTORC1 signaling even under conditions of extensive amino acid deprivation. By restoring mTORC1 activity, aromatic amino acids prevent starvation-induced nuclear export of the proteasome, thereby maintaining its predominantly nuclear localization despite increased cytosolic proteolytic demands. This selective retention alters substrate degradation profiles and, in certain contexts, promotes apoptosis [70] (Figure 4).

Consistent with this model, tumor cells under metabolic stress exhibit enhanced proteasome export to the cytosol, reflecting their dependence on proteolysis for amino acid supply. Conversely, excess availability of aromatic amino acids (YWF) can activate Sestrin3-dependent mTORC1 signaling, promoting nuclear retention of the proteasome, and impairing cytosolic proteolysis required for amino acid recycling. Preclinical studies provide further support for this mechanism. In multiple in vivo models, including xenografts, carcinogens, and genetic manipulation-initiated tumors, and a metastatic breast cancer model in mice, YWF supplementation has been associated with significant inhibition of tumor growth and induction of apoptosis [101]. Notably, YWF administration (delivered either via subcutaneous injection or through drinking water) remains effective even when treatment is initiated at advanced stages, after tumors have reached substantial size. Transcriptomic and proteomic analyses reveal that YWF treatment broadly affects pathways controlling proliferation, migration, and cell death, consistent with a shift toward apoptotic programs. Importantly, these effects appear tumor-selective, as no apparent toxicity or damage has been observed in normal tissues such as the liver or kidney [101].

Taken together, these findings suggest that modulation of amino acid sensing through the Sestrin3-mTORC1 axis represents a potential strategy to disrupt proteasome-dependent metabolic adaptation in tumors, although the precise molecular mechanisms and translational applicability of this approach remain to be better defined.

### 3.4. Proteasome Biomolecular Condensates: p62/SQSTM1-Regulated Condensate Dynamics

In parallel with changes in nucleo-cytosolic proteasome localization, cells deploy an additional adaptive mechanism involving the formation of biomolecular condensates that reorganize proteolytic activity of the proteasome. Among the recently characterized examples are p62/SQSTM1-containing condensates, formed by a multifunctional scaffold protein that coordinates both proteasomal degradation and autophagy [87,102,103,104].

p62 harbors multiple interaction domains that enable multivalent binding. Its Phox and Bem1 (PB1) domain, among other functions, mediates head-to-tail self-oligomerization, whereas its UBA domain binds polyUb chains [105]. Under basal conditions, p62 exists predominantly as dynamic, soluble oligomers [106]. However, stress conditions such as amino acid starvation, oxidative stress, or heat shock promote the accumulation of ubiquitinated substrates, and drive liquid–liquid phase separation of p62 into dynamic condensates through cooperative multivalent interactions between p62 oligomers and polyUb chains [102]. The UBA domain further concentrates ubiquitinated substrates within these assemblies, generating a microenvironment enriched in degradation targets.

The proteasome is subsequently recruited to the surface of p62 condensates, where it degrades ubiquitinated substrates. This recruitment is mediated by interactions involving the Rpn10 subunit of the 19S RP with both: (i) the proteasome-interaction region (PIR) within p62’s PB1 domain [107], and (ii) Ub chains on condensate-associated substrates [108]. By co-localizing substrates and the entire conjugation and degradation machineries within confined spaces, these condensates enhance proteolytic efficiency and facilitate the clearance of proteins that would otherwise accumulate during stress. Experimental measurements have shown increased local degradation rates within such condensates [87,102,103,109].

The functional properties of p62 assemblies depend on their material state. In their liquid-like form, condensates exhibit dynamic behavior, including fusion, fission, and rapid exchange of their components with the surrounding cytosol [102,109,110], thereby enabling continuous and adaptive substrate turnover while preventing irreversible aggregation [111].

Resolution of condensates occurs upon restoration of favorable conditions or through targeted degradation pathways. In some cases, condensate-associated material is selectively removed via autophagy, contributing to the clearance of aggregated proteins and the re-establishment of cellular homeostasis.

### 3.5. RAD23B-Driven Nuclear Condensate Dynamics

In addition to p62 condensates, which can form in both the nucleus and the cytoplasm, nuclear proteostasis is further supported by phase-separated assemblies formed by other Ub shuttle factors, such as RAD23B [112]. This protein contains a UBA domain that binds polyubiquitinated substrates and a UBL domain that mediates interaction with the proteasome, enabling coordinated substrate recognition and delivery [113].

Under stress conditions such as hyperosmotic and acute proteotoxic stresses, RAD23B forms dynamic nuclear condensates that concentrate ubiquitinated nuclear proteins generated during the stress and, in parallel, recruit proteasomes [112]. These substrates are mostly in excess of misassembled nuclear protein complexes, such as unassembled ribosomal subunits and other newly synthesized nuclear proteins that fail to incorporate into functional assemblies. These condensates are thought to facilitate efficient proteolysis of nuclear substrates and support proteasome-mediated nuclear PQC. Importantly, p62- and RAD23B-based condensates arise independently, with RAD23B condensates operating exclusively in the nucleus [102].

Together, coordination between nuclear RAD23B condensates and cytoplasmic p62 bodies provides a pan-cell proteolytic system that enables efficient handling of substrates originating in different locations, particularly under proteotoxic stress conditions, which are also typical of cancer cells.

While these observations underscore the importance of spatial proteasome regulation during cellular stress, several key questions remain unresolved. The molecular mechanisms that coordinate nutrient sensing with proteasome translocation are still incompletely understood, and it remains unclear whether additional nutrient sensors or signaling pathways cooperate with the Sestrin3–mTORC1 axis to regulate proteasome localization. Likewise, the mechanisms governing proteasome recruitment to distinct biomolecular condensates, the determinants of condensate specificity, and the interplay between condensate-mediated proteolysis and proteaphagy require further investigation. Also, as numerous target substrates are ubiquitinated, an open question relates to their recognition. Are they recognized by their “individual” ligases, or is there a “universal” ligase that recognizes them all? The question here is the identity of the common signal that this putative ligase recognizes. Addressing these questions will provide a more comprehensive understanding of how spatial organization contributes to proteasome regulation during cellular adaptation to stress.

### 3.6. Proteaphagy and Proteasome–Autophagy Crosstalk

Proper cellular function and survival under stress rely on efficient PQC, which is maintained primarily by two proteolytic systems: the UPS and the ALP. Beyond their biochemical activities, both systems are dynamically regulated, and their interplay contributes to the spatial organization of proteolysis within the cell. Importantly, UPS is readily available and is recruited a short time after the induction of stress. In contrast, the autophagic system requires more time to become organized, generating its membranous compartment [80].

A key aspect in the UPS–autophagy interplay is the coordinated regulation of their activities. Inhibition of proteasomal activity triggers compensatory upregulation of autophagy, promoting the clearance of accumulated substrates and relieving proteotoxic stress. Conversely, since the proteasome is an abundant cellular protein, under prolonged amino acid starvation, proteasomes are selectively sequestered and redistributed into autophagic compartments for lysosomal degradation, a process termed proteaphagy.

In mammalian cells, this pathway is mediated by the selective autophagy receptor p62/SQSTM1, which recognizes ubiquitinated proteasomes and links them to LC3-decorated autophagosomal membranes [59]. For comparison, in *Arabidopsis*, the proteasome-associated Ub receptor RPN10 performs an analogous targeting function [97].

The regulated re-localization and selective removal of proteasomes establish a spatial feedback mechanism that couples proteasome abundance and activity to cellular stress conditions. By controlling where proteasomes reside within the cell—either as active complexes in the cytoplasm or sequestered within autophagic compartments—cells dynamically tune proteolytic capacity in both space and time.

## 4. 26S Proteasome Dynamics in Disease: From Cancer to Aging

Proteasome regulation is profoundly reshaped in disease, where alterations across structural, post-translational, and spatial levels can disrupt cellular proteostasis. In cancer, proteasome capacity is adaptively enhanced to sustain survival under chronic proteotoxic and therapeutic stress, whereas in neurodegeneration, proteostasis imbalance arises in a context-dependent manner driven by aberrant protein species and cellular stress. In aging, progressive decline in proteasome function further limits protein quality control. Together, these disease contexts illustrate how dysregulated proteasome dynamics contribute to the collapse of proteostasis in pathologic states.

### 4.1. Proteasome Dynamics and Therapeutic Adaptations in Cancer

Cancer cells exhibit extensive remodeling of proteostasis networks to support rapid proliferation and survival under conditions of chronic stress [69,114]. A central component of this adaptation is the proteasome, the activity of which is dynamically regulated through changes in subunit expression, complex assembly, and spatial organization. These adjustments enhance proteolytic capacity and enable tumor cells to cope with increased proteotoxic stress driven by oncogenic signaling, aneuploidy, and elevated protein synthesis [87,102,114,115].

A major mechanism underlying proteasome upregulation in cancer involves the stress-responsive transcription factor NRF1, which under basal metabolic conditions is associated with the ER. Upon proteasome impairment or increased proteotoxic burden, NRF1 is stabilized through reduced degradation and limited proteolytic processing, allowing its translocation to the nucleus. There, it induces transcription of proteasome subunits and assembly factors, promoting de novo proteasome biogenesis and restoring degradative capacity, which is essential for cancer cells to cope with proteotoxic stress [116]. In addition, oncogenic signaling pathways such as mTORC1 can indirectly increase proteasome demand by enhancing global protein synthesis and metabolic activity, thereby intensifying proteotoxic stress and further promoting NRF1-mediated proteasome biogenesis [117].

Cancer cells often exhibit altered expression of proteasome subunits, including PSMB5, to meet their increased demands for protein turnover and adaptation to cellular stress. Elevated PSMB5 expression or activity can enhance proteasome function, enabling tumor cells to maintain proteostasis, tolerate proteotoxic stress, and in some cases, develop resistance to proteasome-targeting therapies. Resistance to proteasome inhibitors such as bortezomib^®^ can also arise through changes in proteasome composition and activity. In certain contexts, altered subunit expression or compensatory increase in proteasome biogenesis allows tumor cells to sustain proteolytic activity despite drug treatment, thereby reducing therapeutic sensitivity [118].

Additionally, in cancer cells, there is enhanced proteasome activity and redistribution of proteostasis capacity toward the cytoplasm, reflecting increased degradation demands from elevated protein synthesis and proteotoxic stress [119]. This is the case, for example, in Multiple Myeloma, where an excess of a single species of an immunoglobulin is synthesized by a B-cell that was clonally expanded. This accelerated synthesis is accompanied by a parallel accumulation of misfolded molecules that are degraded by the proteasome. Inhibition of the proteasome increases the accumulation of the misfolded Ig molecules, resulting in Unfolded Protein Response and apoptosis of the malignant cells. This response is the basis for the efficiency of proteasome inhibitors in the treatment of this malignancy. This cytoplasmic enrichment under conditions where the proteasome is fully active supports the maintenance of proteostasis during high anabolic demand and complements transcriptional programs governing proteasome biogenesis, thereby adding a layer of regulation to proteasome activity in tumors.

### 4.2. Condensate Dysfunction and Proteasome Sequestration in Neurodegeneration

Neurodegenerative diseases such as Huntington’s disease and other protein aggregation disorders are characterized by the accumulation of misfolded, aggregation-prone proteins and progressive disruption of cellular protein homeostasis. In cellular models, expression of polyglutamine (polyQ)-expanded huntingtin, other polyQ-expanded proteins, or unrelated aggregation-prone proteins, has been shown to impair Ub-dependent proteolysis, indicating that proteostatic stress can be induced by aggregation-prone species under defined experimental conditions [120]. However, subsequent mechanistic studies have refined the interpretation of this relationship. In a key experimental study, soluble misfolded or aggregation-prone species were shown to induce a global impairment of UPS function, and importantly, this dysfunction can occur independently of visible inclusion body formation [121]. These findings indicate that inclusion bodies are not required for proteasome dysfunction and argue against a simple model in which large aggregates mechanically obstruct proteasomes. Consistent with this, additional cellular and in vivo studies using UPS reporter systems have shown that proteasome activity can remain largely functional in the presence of visible inclusions or in transgenic models expressing aggregation-prone proteins [122,123]. These observations suggest that overt inclusion pathology is not necessarily synonymous with global proteasome failure.

At the level of intracellular organization, disease-associated proteins such as FUS and polyQ-expanded huntingtin have been shown to undergo liquid–liquid phase separation in reconstituted cellular systems. These proteins can form dynamic condensates that, under certain conditions, transition into less dynamic or fibrillar assemblies, linking phase behavior to aggregation pathways relevant to disease [124,125,126,127]. While these findings establish a mechanistic route from condensates to aggregation, the direct and general sequestration of proteasomes into such assemblies is not consistently demonstrated across systems and remains context-dependent [128].

Together, these experimental data support a model in which neurodegeneration reflects a distributed and context-dependent failure of proteostasis, rather than a single dominant mechanism such as proteasome “clogging.” In this framework, proteasome dysfunction arises primarily from soluble aggregation-prone species, altered protein flux, and stress-induced imbalance of proteostatic networks, while inclusion bodies and condensates represent downstream or modulatory features whose impact varies across models and disease contexts.

### 4.3. Age-Related Impairment of Proteasome Function

Alterations in proteasome regulation during aging involve coordinated changes in expression, assembly, and post-translational modification of proteasome components. Multiple studies in mammalian tissues, yeast, and human primary cells have shown that aging is associated with differential expression of proteasome subunits, including both 20S core and 19S RPs, accompanied by a decline in proteasome activity in several tissues. These changes correlate with reduced proteolytic capacity and altered proteasome composition in aging cells. In particular, impaired proteasome structure and function have been demonstrated in aged systems, while reduced proteasome activity has been shown to contribute to a senescence-like phenotype in human fibroblasts [129,130]. In parallel, age-related decline in proteasome biogenesis has been linked to reduced efficiency of assembly pathways that depend on dedicated chaperones, including PAC and UMP1/POMP-related factors, resulting in decreased formation of fully mature and functional 26S proteasome complexes [131,132].

In addition to changes in abundance and assembly, proteasome complexes themselves accumulate biochemical damage with age. Experimental analyses of aged tissues have demonstrated age-dependent alterations of proteasome subunits together with increased accumulation of oxidatively modified proteins, which correlate with reduced catalytic activity and impaired proteasome function [133,134,135]. Oxidative modifications can affect both the structural integrity and peptidase activities of proteasome complexes, particularly under conditions of chronic oxidative stress [133,134,135,136].

Additionally, it is well-established that aging cells accumulate increased levels of oxidatively modified, cross-linked, or otherwise structurally damaged proteins, which are poor substrates for proteasomal degradation. Biochemical and cell-based studies have shown that such oxidized proteins accumulate over time and are degraded less efficiently than native substrates, thereby increasing the burden on the UPS [137,138,139]. This shift in substrate quality contributes to reduced effective proteolytic throughput, even when functional proteasome complexes remain present.

Together, these experimentally observed changes indicate that the age-related decline in proteasome function stems from multiple factors. These include changes in abundance, assembly efficiency, and biochemical integrity, combined with an increased load of damaged proteins that are less efficiently processed by the degradation machinery.

## 5. Summary

The dynamic regulation of proteasome function represents a central mechanism by which cells maintain proteostasis across diverse physiological conditions. The integration of spatial localization, nutrient signaling, and biomolecular condensation enables an adaptable system capable of responding to changes in metabolic demands and environmental stress.

A key insight emerging from recent studies is that proteostasis regulation is not governed solely by global nutrient availability but can be modulated by specific amino acid signals. In particular, Gln, Leu, and Arg (QLR)-dependent regulation of mTORC1 mediated via Sestrin1/2 and YWF-dependent regulation of mTORC1 mediated via Sestrin3 highlight how nutrient composition can affect cellular metabolic and proteostatic states. This introduces an additional layer of specificity that may contribute to context-dependent regulation of proteostasis networks, including proteasome activity.

At the same time, biomolecular condensates provide a spatial context for coordinating proteolytic processes. Phase separation enables the local concentration of substrates and proteostasis factors, thereby enhancing reaction efficiency without the requirement for membrane-bound compartments. The dynamic properties of these assemblies support adaptability, whereas altered material states can compromise their regulatory function.

The interplay between the UPS and autophagy further illustrates the multilayered nature of proteostasis regulation. These pathways are functionally interconnected through shared substrates, signaling networks, and quality control mechanisms. Disruption of this coordination can lead to imbalances in protein degradation capacity and contribute to proteostasis stress. A summary of the varied factors associated with the regulation of the proteasome is provided in Table 1.

## Figures and Tables

**Table 1:** Major regulators of proteasome dynamics and their functional consequences.

Regulator	Main Compartment	Trigger	Effect on Proteasome	Functional Outcome	References
mTORC1	Lysosome-associated signaling hub	Nutrient-rich conditions	Promotes nuclear proteasome retention and anabolic state	Regulation of metabolism, cell growth, and survival	[59,94,95,96,97]
Sestrine3	cytosol	Aromatic amino acid limitation	Inhibits mTORC1, stimulates proteasome export	Reprograms proteostasis during starvation	[70,101]
p62/ SQSTM1	Cytosolic condensates	Starvation, proteotoxic stress	Recruits ubiquitinated substrates and proteasomes into phase-separated bodies	Enhances local degradation and proteaphagy	[87,102,103,109]
Rad23	nucleus	Acute stress (hyperosmotic or proteotoxic)	Forms nuclear condensates with ubiquitinated proteins and proteasome	Promotes nuclear protein quality control	[112]
NRF1	ER and nucleus	Proteasome impairment	Induces expression of proteasome subunits and assembly factors	Restores proteasome capacity	[116,117]
CRM1/ exportin1	Nucleus/cytosol	Starvation or stress	Mediates proteasome nuclear export	Redistributes proteolytic capacity to the cytosol	[70]
AKIRIN2	Nucleus/cytosol	Nutrient-rich conditions	Facilitates nuclear import	Redistributes proteolytic capacity to the nucleus	[74]
19S/PA700	Nucleus/cytosol	Nutrient-rich conditions	Ensures binding, deubiquitination, unfolding, and translocation of substrates	Turnover of key factors in cellular regulation in an ATP- and Ub-dependent manner	[28]
PA28	Nucleus/cytosol	Specialized immune and stress settings	Enhanced peptide cleavage and substrate processing	Supports antigen processing and stress adaptation	[43,44]
PA200	Nucleus/cytosol	Nuclear and chromatin-associated contexts	Promotes specialized proteasome activity	Supports histone turnover and nuclear proteostasis	[21,45]

## Data Availability

No new data were created or analyzed in this study. Data sharing is not applicable to this article.

## Abbreviations

ALAD	δ-aminolevulinic acid dehydratase
ALP	autophagy-lysosome pathway
Arg, R	arginine
CCR	catalytic core regulators
CP	core particle
cryo-EM	cryo-electron microscopy
DUB	deubiquitinase
ER	endoplasmic reticulum
ERAD	ER-associated degradation
FAD	flavin adenine dinucleotide
iCP	immunoproteasome
Leu, L	leucine
LLPS	liquid-liquid phase separation
Lys, K	lysine
NLS	nuclear localization signal
NPC	nuclear pore complex
NQO1	NAD(P)H dehydrogenase [quinone] 1
ODC	ornithine decarboxylase
PB1	Phox and Bem1
Phe, F	phenylalanine
PI31	proteasome inhibitor subunit 1
PIR	proteasome-interaction region
polyQ	polyglutamine
PML	promyelocytic leukaemia
PQC	protein quality control
PSGs	Proteasome Storage Granules
RP	regulatory particle
tCP	thymoproteasome
Trp, W	tryptophan
Tyr, Y	tyrosine
Ub	ubiquitin
UBA	ubiquitin-associated
UBL	ubiquitin-like
UBR	ubiquitin- binding receptor
UCH-L5	Ub carboxyl-terminal hydrolase isozyme L5
UPS	ubiquitin–proteasome system
USP14	Ub-specific peptidase 14
USP15	Ub-specific peptidase 15
USP7	Ub-specific peptidase 7
